# The role of tumor types in immune-related adverse events

**DOI:** 10.1007/s12094-024-03798-6

**Published:** 2024-12-30

**Authors:** Qian Xu, Jing Hu, Yan Wang, Zhaohui Wang

**Affiliations:** 1 Department of Cardiology, Union Hospital, Tongji Medical College, Huazhong University of Science and Technology, Wuhan, 430000 Hubei China; 2 Hubei Key Laboratory of Biological Targeted Therapy, Union Hospital, Tongji Medical College, Huazhong University of Science and Technology, Wuhan, 430000 Hubei China; 3 Hubei Provincial Engineering Research Center of Immunological Diagnosis and Therapy for Cardiovascular Diseases, Union Hospital, Tongji Medical College, Huazhong University of Science and Technology, Wuhan, 430000 Hubei China; 4 Department of Infectious Diseases, Union Hospital, Tongji Medical College, Huazhong University of Science and Technology, Wuhan, 430000 Hubei China

**Keywords:** Biomarker, Immune-checkpoint inhibitors, Immune-related adverse events, Pathogenesis, Tumor-heterogeneous

## Abstract

Immune checkpoint inhibitors (ICIs) are monoclonal antibodies that block inhibitors of T cell activation and function. With the widespread use of ICIs in cancer therapy, immune-related adverse events (irAEs) have gradually emerged as urgent clinical issues. Tumors not only exhibit high heterogeneity, and their response to ICIs varies, with “hot” tumors showing better anti-tumor effects but also a higher susceptibility to irAEs. The manifestation of irAEs displays a tumor-heterogeneous pattern, correlating with the tumor type in terms of the affected organs, incidence, median onset time, and severity. Understanding the mechanisms underlying the pathogenic patterns of irAEs can provide novel insights into the prevention and management of irAEs, guide the development of biomarkers, and contribute to a deeper understanding of the toxicological characteristics of ICIs. In this review, we explore the impact of tumor type on the therapeutic efficacy of ICIs and further elucidate how these tumor types influence the occurrence of irAEs. Finally, we assess key candidate biomarkers and their relevance to proposed irAE mechanisms. This paper also outlines management strategies for patients with various types of tumors, based on their disease patterns.

## Introduction

Tumor cells express specific molecules on their surfaces that can inhibit the activity of immune cells [1–4], enabling them to evade immune system attacks. Immune-checkpoint inhibitors (ICIs) are monoclonal antibodies that counteract immune evasion by blocking these molecules, reactivating immune recognition and attack against tumors [5–7]. However, adverse events triggered by ICIs may interrupt their therapeutic effects and significantly affect patient prognosis [8]. Immune-related adverse events (irAEs) can occur in any organ, ranging from transient and undetectable side effects to highly morbid, occasionally permanent [9], and even fatal events [10]. Identifying the factors that influence these differences will facilitate the development of predictive biomarkers, identification of patients at risk of developing irAEs, and optimization of cancer treatment. The incidence of irAEs may be affected by patient-specific factors, tumor characteristics, and the type of ICIs used.

Although the type of ICI administered is believed to influence irAE occurrence [11–13], clinical observations challenge this assumption. Patients receiving the same ICI can experience different irAE characteristics, suggesting a potential role for tumor type [14, 15]. Tumors are recognized as new growths resulting from sequential genetic changes and clonal expansions, showing a range of mutations and biologic characteristics [16]. Evidence suggests that genetic and phenotypic differences exist both between different tumor types and among patients with the same cancer type. These differences are associated with how the tumor responds to drug treatment [17]. However, the specific relationships between tumor types and irAEs, as well as their underlying mechanisms, remain underexplored and poorly defined. Investigating tumor-related factors may provide a novel perspective that complements the triangular relationship between patients, tumors, and ICIs. This approach can offer a more comprehensive explanation for the occurrence patterns of irAEs.

This review aims to examine how different tumor types influence various aspects of irAEs. Firstly, we analyze variations in ICI treatment responses among different tumor types to identify those more likely to induce irAEs. Subsequently, we delve into how these tumor types influence the occurrence patterns of irAEs and investigate their potential biologic mechanisms or risk factors. Finally, we introduce existing predictive biomarkers or risk management strategies based on known mechanisms and risk factors that are expected to aid in identifying patients with a higher risk of irAEs and optimizing their cancer treatment strategies.

Through this review, we hope to provide clinicians and researchers with a comprehensive and in-depth perspective to prospectively identify and manage patients at risk of irAEs.

## Differences in therapeutic responsiveness

In contemporary oncology, ICIs, particularly those targeting PD-1 and PD-L1, have gained regulatory approval for the treatment of various malignancies [18–20]. PD-1 is a key immunosuppressive checkpoint primarily expressed in immune cells such as macrophages, B lymphocytes, dendritic cells, monocytes, activated T cells, myeloid cells, and natural killer (NK) cells during chronic antigen exposure [21–23]. PD-L1, serving as a ligand for PD-1, is present in tumor cells, tumor-infiltrating cells, and antigen-presenting cells across a spectrum of cancers [21, 24]. They function in peripheral tissues during the effector phase of the immune response through their mutual binding, shutting down the immune response and preventing potential autoimmune damage following prolonged antigen exposure [25]. Tumors can exploit this pathway to suppress the local host immune response against them. In this mechanism, surveillance T cells recognize tumor neoantigens as foreign and become activated, upregulating PD-1 and secreting interferon-γ (IFN-γ). In response to IFN-γ, tumor cells and immune cells in the immediate tumor microenvironment express PD-L1. Subsequently, PD-L1 binds to PD-1, effectively deactivating the surveillance T-cells [26, 27]. These ICIs have achieved remarkable success in treating certain solid tumors, such as those associated with lung cancer and melanoma [20, 28]. However, only about one-third of patients respond favorably to these therapies, hindering the efficacy of ICIs [29].

**Fig. 1:**
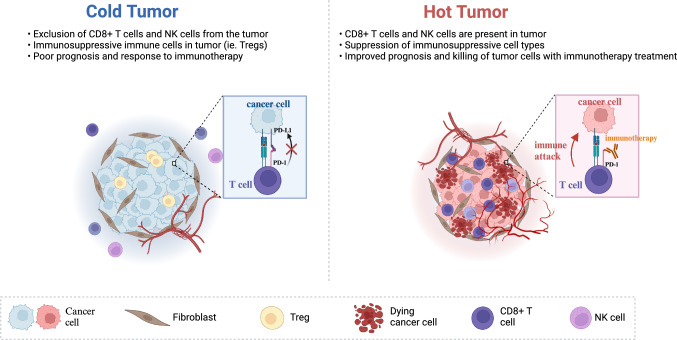
Tumor immunophenotypes. Based on the distribution and abundance of cytotoxic immune cells in the tumor microenvironment (TME), tumors can be classified into immunogenic tumors, also known as “hot tumors”, or immunologically excluded tumors (“cold tumors”). Hot tumors are characterized by robust antigen release, significant T-cell infiltration, and an inflammatory microenvironment, often exhibiting good responses to immune check point inhibitors (ICIs). In contrast, immunologically excluded tumors lack CD8^+^ T cells in both the tumor core and periphery, suppressing the body’s anti-tumor immune response, resulting in poor therapeutic efficacy of ICIs

Tumors are complex heterogeneous tissues that exhibit diverse biologic and genetic abnormalities resulting from abnormal cell invasion and growth during their formation and development [30]. These abnormalities are not only confined to the tumor cells themselves, but also involve complex interactions within the entire tumor microenvironment (TME). The responsiveness of tumors to treatment largely depends on their genetic mutations and activated signaling pathways [31]. The TME plays a crucial role in immune responses, and tumors are further classified into different immune phenotypes based on the distribution and status of immune cells within the TME (Fig. 1), including immune-inflammatory (“hot” tumors), immune-exclusive, and immune-desert (“cold” tumors) types [7]. These different tumor types exhibit significant differences in their responses to immunotherapy. Immune-inflammatory tumors typically demonstrate high T cell infiltration, robust IFN-γ signaling, PD-L1 expression, and a high tumor mutational burden [32]. These characteristics make “hot” tumors well-suited for immunotherapy using ICIs and represent important targets for immune therapy [33, 34]. Conversely, in immune-exclusive and immune-desert tumors, the efficacy of immunotherapy is significantly reduced owing to limited or absent infiltration of CD8^+^ T lymphocytes [35, 36].

Based on the understanding that “hot” and “cold” tumors exhibit different responses to treatment, clinical observations have shown that “hot” tumors, such as those associated with breast cancer, melanoma, non-small cell lung cancer (NSCLC), renal cell carcinoma, and gastric cancer, are more likely to respond positively to immunotherapy. Given that ICIs may lead to irAEs and tumor regression by enhancing immune responses, studies have noted a positive correlation between these occurrences. For example, irAEs were associated with tumor regression in patients with metastatic renal cell carcinoma(mRCC) or melanoma treated with ipilimumab [34–37]. “Hot” tumors may exhibit a higher propensity for irAE development compared to “cold” tumors. This review aims to explore how different types of “hot” tumors contribute to distinct patterns of irAEs, shedding light on the relationship between tumor heterogeneity and adverse reactions. By doing so, it aims to provide a valuable reference for clinicians and researchers.

## Patterns of tumor heterogeneity in irAEs

The spectrum of irAE presentations ranges from mild, transient side effects to severe, occasionally persistent, and life-threatening conditions. Variations exist in terms of affected organs, incidence rates, severity, and onset time. This section aims to synthesize current clinical data and examine the role of tumor types from various perspectives, offering a comprehensive understanding of how tumor types influence irAEs.

### Organ heterogeneity

**Fig. 2:**
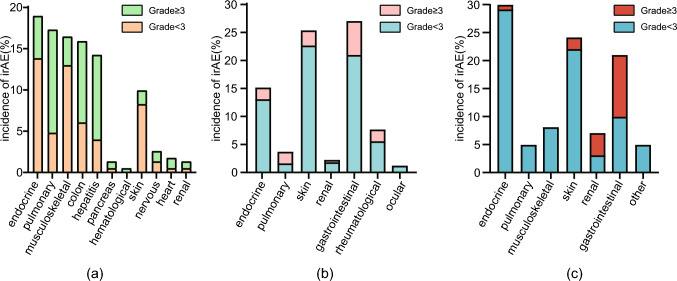
The occurrence of irAEs varies in terms of organs and grades among different tumor types [38–40]. **a** Grade distribution of irAEs observed in each organ for NSCLC patients. **b** Grade distribution of irAEs observed in each organ for melanoma patients. **c** Grade distribution of irAEs observed in each organ for mRCC patients

One explanation for the organ-specificity of irAEs is that different mono- or combination therapies may predispose patients to specific types of adverse events. However, even with the same treatment, different patients may exhibit different irAEs, suggesting that additional individual differences play a role. Tumor type in the primary organ is a crucial factor influencing the incidence of irAEs (Fig. 2). For instance, in patients with melanoma treated with PD-1 inhibitors, skin-related irAEs (particularly vitiligo) and gastrointestinal irAEs are more common. In contrast, patients with renal cell carcinoma tend to manifest respiratory system-related irAEs such as pneumonia and dyspnea [14]. Another study using PD-1 inhibitors revealed that patients with multiple myeloma often exhibited more diarrhea, skin issues, endocrine (hypothyroidism), and musculoskeletal (joint pain) issues, but fewer pulmonary issues (pneumonia and dyspnea) than patients with NSCLC and renal cell carcinoma [41]. CTLA-4 inhibitors are typically used for melanoma or mixed tumors, rendering similar analyses comparing tumor-specific irAE profiles infeasible. Despite the limited data, these studies suggest that different tumor types can drive distinct irAE patterns in various organs. Interestingly, irAEs may exhibit differences even within the same organ [42–44]. Cardiovascular irAEs are frequently reported in patients with melanoma or lung cancer, with prospective studies showing that patients with lung cancer most commonly experience pericardial disease [45], whereas patients with melanoma have higher rates of myocarditis and vasculitis [46]. Although ICIs are more prevalent in melanoma and lung cancer treatment, potentially leading to over-reporting of specific malignancies, the underlying mechanisms behind this variation require further elucidation.

### Incidence

The overall incidence of irAEs is also closely associated with tumor type. Specifically, patients with melanoma and gastric cancer have high overall irAE incidence rates (37.5% and 12.5%, respectively) when undergoing immunotherapy, indicating their high sensitivity to this treatment. In addition, the incidence rates remain high, regardless of the administration of ICIs alone or in combination, further emphasizing the significant role of tumor type in the occurrence of irAEs. In contrast, patients with gynecological cancer exhibit relatively lower irAE incidence rates (7.5%), although this is not insignificant. Similarly, the incidence rates of lung cancer and lymphoma stand at 5% [42]. Wang et al. conducted a real-world observational data analysis comparing the irAE risks in seven cancer types between patients receiving nivolumab or pembrolizumab PD-1 inhibitors and those undergoing chemotherapy or targeted therapy [47]. Their analysis revealed that, compared with matched chemotherapy or targeted therapy groups, patients with head and neck cancers had the highest risk of irAEs, whereas those with brain cancer exhibited the lowest risk. Similar outcomes were observed in patients treated with pembrolizumab.

These findings may stem from the different response patterns of various tumor types to immunotherapy. It should be noted that deciphering the correlation between irAE incidence and tumor type remains challenging, with various studies yielding conflicting results. A large meta-analysis of more than 20,000 patients from 125 clinical trials revealed similar irAE incidence rates across all cancer types [48]. Analyzing the reasons for these contrasting conclusions, we postulate that differences in patient populations, treatment protocols, and follow-up durations among studies may contribute to the variations in irAE incidence and manifestation patterns.

Moreover, discrepancies in the definitions, assessment criteria, and diagnostic methods of irAEs among studies may exist. Such variations could lead to an underestimation or overestimation of irAE incidence, thereby affecting the consistency of the research conclusions. To more accurately assess the association between irAE incidence and different tumor types, it is crucial to conduct high-quality, large-scale clinical studies and enhance the monitoring and management of irAEs.

### Median time and severity

**Fig. 3:**
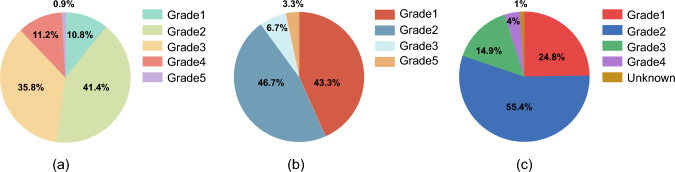
Grade distribution of irAEs observed in different types of tumors [38–40]. **a** Grade distribution of irAEs observed in NSCLC patients. **b** Grade distribution of irAEs observed in melanoma patients. **c** Grade distribution of irAEs observed in mRCC patients

Tumor type also influences the median time and severity of irAEs (Table 1, Fig. 3). A meta-analysis published in 2020, encompassing 11 trials, reported that fatal adverse events related to pembrolizumab were highest in patients with breast cancer, reaching 1–3.2%, followed by those with NSCLC at 2%, gastric cancer at 0.8%, and melanoma at 0.2% [49]. Another study involving 4496 patients revealed that compared to melanoma, NSCLC had a higher incidence of pneumonitis of all grades 3 or above, while renal cell carcinoma showed an increased incidence of pneumonitis across all grades, albeit without an increase in events of grade 3 or above [50]. Unlike the toxicities induced by cytotoxic or molecular targeted drugs, the onset of irAEs triggered by ICIs does not follow a cyclical pattern similar to those of traditional cytotoxic drugs and may be influenced by multiple factors. In ICI-induced pneumonitis, the median onset time was 3.8 months for patients with NSCLC and 14.6 months for patients with malignant melanoma [39, 51, 52]. When comparing the onset time of irAEs between multiple patients with myeloma and lung cancer, it was found that the onset of irAEs in patients with multiple myeloma patients occurred later than in patients with lung cancer, with median times of 5.2 months and 2.1 months, respectively. These findings may vary across different studies, indicating the need for further research to achieve validation [41].

**Table 1:** Time to onset of immune-related adverse events (median with range) in different tumors (weeks) [46–48]

irAEs	Melanoma	NSCLC	mRCC
Pulmonary	62.4	16.2(1.6–108)	40.1 (3–62.1)
Hepatic	24.0	8.7(0.1–131)	1.4
Skin	12.0	6.1(0.1–100)	3.2 (2.1–41.1)
Gastrointestinal	21.6	4.9(0.1–91)	23.2

## Pathogenesis

The mechanisms underlying irAEs are complex and diverse, and there is currently a lack of well-established evidence. Various hypotheses have been proposed to explain these underlying biologic processes. The first hypothesis states that because ICIs exert their effects by activating the immune system, the disruption of self-immune tolerance and promotion of pre-existing autoimmunity contribute to the occurrence of irAEs [53]. The second hypothesis, known as the shared antigen theory, aims to explain the immune recognition of ICIs in healthy tissues due to the presence of antigens similar to those in tumor tissues [53–56]. Similarly, the antigen mimicry hypothesis suggests that T cells targeting tumors can also target wild-type antigens in other organs [53]. In addition, inflammatory mediators, such as cytokines and chemokines, produced during immune responses may damage tumor-adjacent tissues, leading to autoimmunity [53, 57]. Another hypothesis suggests that the use of ICIs generates antibodies targeting immune checkpoints, and that some irAEs may be caused by the off-target effects of these antibodies. For example, ICIs can target the hypothalamic and pituitary tissues expressing CTLA-4, leading to hypophysitis [53]. Moreover, microbiome studies have suggested that they play a crucial role in promoting or preventing irAEs by mediating the production of pro-inflammatory or anti-inflammatory cytokines [54, 58–61].

Furthermore, tumor type can serves as an independent predictor of irAEs and participate in risk stratification [42]. This section elaborates on the potential mechanisms mediating the heterogeneous patterns of irAEs from the perspective of differences in different tumors, including differences in tumor types and subtypes.

### Tumor mutation burden

A large study using data from the FAERS database involving over 16,000 patients with irAE reported that tumor mutation burden is a significant risk factor for irAEs across multiple cancer types [62]. A higher median number of somatic mutations per megabase of DNA is associated with a greater risk of irAEs.

### Tumor-shared antigen epitopes

One of the aforementioned hypotheses for the development of irAEs is the shared antigen theory, which is based on the interesting fact that patients with irAEs (with activated anti-tumor responses) tend to have improved responses to ICI treatment and better survival rates [63–67]. According to this theory, antigens that mediate anti-tumor responses in patients with cancer are also considered mediators of irAEs [54]. Many studies have reported this observation, suggesting that many irAEs are driven by specific responses to primary tumors [68].

A notable example involves patients with melanoma. Melanoma and melanocytes share the same cell type, and melanocyte differentiation antigens are co-expressed in tumor tissues, the epidermal layer of the skin, and melanocytes in hair follicles. This dual presence of antigens can mediate effective anti-tumor responses, but may also lead to the occurrence of skin irAEs such as vitiligo [69, 70]. The shared antigen theory still applies when discussing irAEs in patients with lung cancer. Napsin A, an aspartic protease, is typically expressed in the type II alveolar cells of lung parenchyma. Some studies have reported that it mediates the occurrence of pneumonitis in NSCLC. Conversely, in patients with melanoma, the stimulation of T cells with napsin A produces minimal T cell responses, indicating that napsin A-mediated T cell responses are specific to patients with NSCLC. Correspondingly, high-grade and all-grade pneumonitis occur most frequently in patients with NSCLC [71]. Notably, because napsin A is expressed at higher levels in lung adenocarcinoma than in lung squamous cell carcinoma (SCC), it is likely more involved in adenocarcinoma-related rather than SCC-related responses. Following the lungs, the skin exhibits the second-highest histologic similarity to NSCLC, thus contributing significantly to autoimmune adverse reaction.

In cardiac irAEs, the shared antigen mechanism of common T-cell clone recognition between tumors and anatomic sites has also been demonstrated [72, 73]. In fact, common T-cell clones have been identified in both patient tumors and heart tissues, pointing toward the possibility of these clones being activated at both sites. Common α-myosin-specific T-cell receptors have been found in blood and inflamed heart tissues [74]. These data suggest that α-myosin may serves as an important shared autoantigen between tumor and heart tissues, mediating autoimmune responses in the heart. Other studies have identified β-adrenergic receptors as candidate autoantigens mediating ICI-induced myocarditis [74, 75]. Further research is required to determine the role of these autoantigens in the development of myocarditis and to better understand the pathogenesis of this irAE.

To date, the shared antigen theory has only been confirmed in a limited number of irAEs, including skin irAEs, pneumonitis, and myocarditis. However, it may also be one of the mechanisms underlying other irAEs, although further studies are needed to confirm this hypothesis.

### Impact of tumor-mediated peritumoral inflammation

Tumors can induce an inflammatory state in surrounding tissues. ICI-induced immune infiltration can further enhance peritumoral inflammation, resulting in different toxicity patterns depending on the tumor location. This paradoxical exacerbation can be viewed as a focal immune reconstitution inflammatory syndrome, similar to that described in patients with human immunodeficiency virus, in which restoration of the host’s ability to mount an inflammatory response to persistent microbial antigens or autoantigens leads to symptomatic manifestations [76]. Therefore, healthcare providers should be particularly vigilant regarding tumors in lung or brain tissues, as ICI may more readily detect tumor infiltration in these areas. This detection could potentially lead to the development of pulmonary lymphangitis or carcinomatous meningitis, manifesting as dyspnea or headache and often diagnosed as interstitial pneumonia or meningitis [77].

Tumors can also cause inflammation in anatomically adjacent tissues. Patients with lung cancer have a higher incidence of pericarditis due to the proximity of the lungs and pericardium. Some researchers believe that pericarditis is a common side effect of lung cancer regardless of treatment [78]. Tumors can also cause inflammation in distant organs via metastasis. A case report found that microscopic melanoma deposits in inflamed gastric tissue are associated with ICI-related gastritis [79]. However, it remains unclear whether this symptom is caused by inflammation targeting metastatic tumor deposits or by the discovery of other autoantigens that trigger tolerance breakdown, leading to irAEs.

### Prior-treatment modalities and medical history

Prior-treatment modalities and medical history play important roles in explaining why patients with lung cancer have a higher incidence of pneumonia. 

Some studies have found that, compared to patients with melanoma patients, patients with NSCLC and a history of prior lung radiation therapy may experience a higher incidence of pneumonia when treated with PD-1 inhibitors [80–82]. This may be because of the radiosensitivity of the lungs caused by radiation therapy, as radiation-induced pneumonia is common. However, this does not completely explain the high incidence of pneumonia in patients with renal cell carcinoma. This difference suggests an interesting possibility that the effects of prior treatment modalities may vary among different tumor types and ICI classes.

Prior lung disease and smoking history in patients with lung cancer are also risk factors for pneumonia. Multiple retrospective clinical studies have revealed a correlation between smoking and pneumonia development. Smoking history is an independent and influential prognostic factor for checkpoint inhibitors pneumonitis(CIP), leading to non-autoimmune inflammation [83, 84]. Prior lung diseases, such as chronic obstructive pulmonary disease, can weaken the ability of the lungs to resist damage before ICI treatment and promote the recruitment and activation of neutrophils, CD4^+^ and CD8^+^ T cells, B cells, dendritic cells, and macrophages, thereby establishing an inflammatory environment that exacerbates ICI-induced damage.

Patients with lung SCC exhibit a higher susceptibility to pneumonia compared to other subtypes, rendering it a notable risk factor. In an evaluation of 87 patients with CIP, Lin et al. found that SCC subtype and ICI monotherapy were independently and significantly associated with CIP occurrence [85]. This association can be attributed to the increased risk of CIP due to obstructive pneumonia, which is more common in central lung cancers such as SCC.

### Tumor burden

Disease burden also appears to play a role in the incidence of irAEs. Studies have shown that patients with multiple myeloma and lung cancer who have two or more metastatic sites have a higher risk of developing severe irAEs [86]. In a small retrospective evaluation of patients with NSCLC (*n* = 42), those with a high tumor burden had a greater incidence of irAEs [87].

### Gut microbiota

Commensal microorganisms, collectively referred to as microbiota, have been shown to influence human immune responses in both healthy and diseased states [88, 89]. In addition, the diversity and composition of the gut microbiota can affect immunotherapeutic responses in mice and humans [88, 90]. For example, a comparison of the gut microbiota composition in 65 patients with immunotherapy-related colitis revealed enrichment of Prevotella from the Bacteroidetes phylum in patients with severe diarrhea, suggesting that the irAE spectrum is influenced by gut microbiota [91]. Interestingly, a higher diversity and relative abundance of taxa from the phylum Firmicutes may protect against irAEs. Moreover, the size, number, and stage of tumors can influence the distribution of these microbes. Specifically, patients with larger tumors (≥ 5 cm) exhibited a significantly higher abundance of Eubacterium hallii (a member of Firmicutes) than those with smaller tumors (< 5 cm). In addition, patients with a higher abundance of tumor size-positive, number- positive, and stage-positive-related taxa exhibited better survival rates.

## Biomarkers

**Fig. 4:**
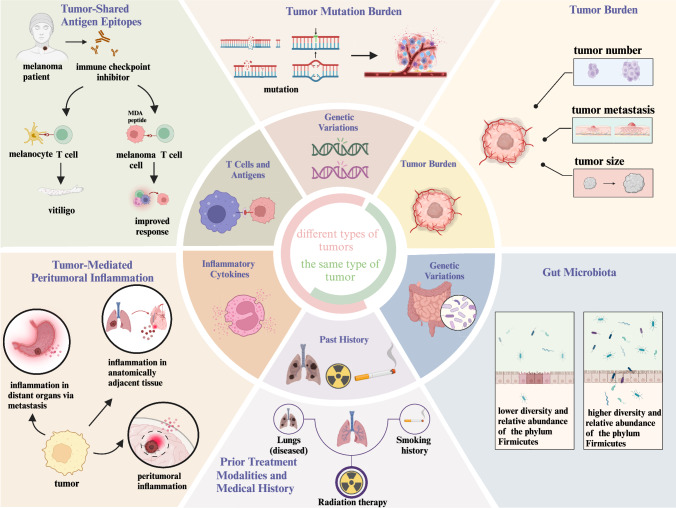
Heterogeneous Patterns and Biomarkers of irAEs Linked to Tumors. Multiple mechanisms may underlie the heterogeneous patterns of irAEs Linked to tumors. Based on these mechanisms, corresponding biomarkers can be developed to prospectively identify individuals with a high risk of adverse events

As the clinical use of ICIs in cancer treatment expands, accompanied by a rise in irAEs, the identification of biomarkers for irAE occurrence has become imperative. The primary objective is to assess the risk of irAEs either prior to initiating ICI therapy or soon after its commencement. Here, we analyze and review key candidate biomarkers, aiming to elucidate their relevance to the proposed mechanisms of irAEs (Fig. 4).

### Specific T cells and corresponding antigens

Given the mechanism of shared antigens, particular attention should be directed toward tissues that share antigens with the corresponding tumors when utilizing ICIs, as these tissues and organs exhibit a heightened risk of irAE development. Consequently, appropriate monitoring strategies should be implemented during treatment. Nevertheless, our understanding of the antigens shared between most tumor types and irAEs remains limited. To address this gap, researchers developed a novel algorithm called DITAS [55]. This algorithm identifies candidate tumor antigens based on computational analysis of transcriptional similarities between tumors and healthy tissues, and combines the latter with functional T-cell assays and single-cell RNA sequencing to validate the identified antigens. As new antigens continue to be unearthed, they will facilitate the establishment of more comprehensive tumor-tissue correspondence risk models, enabling early risk identification. The most useful and feasible biomarkers in clinical settings are often blood-based. Thus, the risk of developing specific irAEs can be predicted by detecting the frequency of specific T cells against corresponding antigens in the blood of patients before treatment.

Moreover, the presence of specific immunoglobulin G against these antigens can also serve as a predictive marker [92]. For example, test results have indicated that patients with NSCLC who developed skin irAEs exhibited elevated anti-BP180 immunoglobulin G (an antibody against melanocyte differentiation antigens) levels before treatment compared to patients who did not develop such irAEs.

### Inflammatory cytokines

Monitoring the expression of inflammatory cytokines is essential for identifying and treating tumors or metastases at specific locations. The inflammatory profile before or early in treatment serves as a baseline, correlating not only with the anti-tumor response post-ICI treatment but also with the potential onset of irAEs [93]. Tissues located close to the anatomic location of the tumor may be more susceptible to the effects of ICI treatment. Increases in inflammatory cytokines, such as IFN-γ, IFN-α, and IL-6, and relative suppression of regulatory cytokines, such as IL-10, IL-35, and TGF-β, may serves as useful predictive biomarkers [94].

### Gastrointestinal microbiota

Gut microbiota regulate the baseline state of the immune system through direct microbiota-immune cell interactions and other complex mechanisms, including the production of metabolites, ultimately playing a role in regulating homeostatic barrier immune profiles. Alterations in the composition of the gut microbiota, known as dysbiosis, are associated with autoimmune diseases and cancer [90, 95–99]. Certain microbiota features are associated with the development of baseline inflammatory characteristics of the gastrointestinal epithelial barrier, ultimately resulting in systemic effects. It is hypothesized that disruptions in immune regulatory mechanisms, such as those induced by ICI treatment, may lead to the promotion of autoimmune phenomena, including irAEs, by certain microbiota. Specific intestinal microbiota, such as the Bacteroidetes-enriched profile, have been identified as both risk factors and biomarkers for irAE development. Researchers have identified various features of the intestinal microbiome associated with an increased risk of irAEs [100–102]. This extensively studied association has been used to predict the incidence of irAEs. In summary, leveraging microbiota composition as a predictive tool for irAEs in patients undergoing ICIs treatment appears to be a promising direction.

### Past history

A previous history of radiotherapy, chemotherapy, smoking, and diseases in corresponding locations can lead to non-autoimmune inflammation, thereby increasing the risk of irAEs. In addition, tumor subtypes carry different irAE risks, and patients with heavier tumor burdens should be more cautious when determining the use of ICIs. Therefore, patients with cancer should be stratified and managed according to the potential risk factors before initiating ICIs treatment. A comprehensive physical examination of patients and a systematic review of their demographic characteristics (including age [46], sex, race, smoking status [103, 104], etc.), medical history (previous diseases and autoimmune diseases) [105–108], and pharmacological history (previous chemotherapy, chest radiotherapy, etc.) are recommended to identify high-risk individuals and devise appropriate treatment plans [92].

### Genetic variations

These mechanisms are closely related to genetic information at a deeper level, which can be traced back to differences and characteristics at the genetic level. Therefore, genetic information holds promise as a comprehensive indicator capable of predicting various pathogenic processes, serving as a potential predictive marker. Several studies have evaluated the role of specific single-nucleotide polymorphisms (SNPs) [109, 110]. Abdel-Wahab et al. identified up to 30 SNPs that were significantly associated with irAEs in patients with melanoma [111]. Twelve SNPs, including those affecting GABRP, DSC2, SEM5A, OSBPL6, and AGPS, increased the risk of irAEs. In contrast, 18 genes were associated with a reduced risk, including RGMA, ANKRD42, PACRG, FAR2, and ROBO1. With the development of genome sequencing technology, an increasing number of SNPs are expected to be discovered and validated, potentially becoming routine pretreatment screening items.

## Management

Managing patients undergoing ICIs treatment requires interdisciplinary collaboration among oncology, immunology, and intensive care medicine. The multidisciplinary team should adopt a balanced approach to minimize toxicity to other organs while limiting the reduction or discontinuation of anticancer treatment. Based on the mechanisms and existing biomarkers discussed, this review summarizes the management strategies for patients with different types of tumors in the following aspects. For tumors with a high mutation burden, such as melanoma and lung cancer, patients are at a high risk of developing irAEs and should be classified into a high-risk irAE cohort. These patients require strict monitoring and management under current practices. When considering which specific organs are affected by irAEs, the discovery of an increasing number of tumor-tissue-specific risk models and a deeper understanding of the risks associated with tumors in adjacent organs, special locations, and different tumor subtypes can guide focused monitoring of organs prone to irAEs, rather than undirected whole-body management. For patients with a history of radiotherapy, chemotherapy, or specific medical conditions, particular attention should be paid to the lungs and heart, with corresponding examinations conducted as necessary. If the tumor metastasizes, progresses in staging, or changes in size, indicating an increased tumor burden, it is essential to monitor inflammatory markers, which may reflect a heightened risk of irAEs. Monitoring of the intestinal microbiota should also be performed. If possible, dietary adjustments, probiotics, or fecal microbiota transplantation can be employed to improve the microbiota environment and reduce the risk of irAEs.

## Discussion

The role of ICI treatment is to improve both the quantity and quality of life for patients. However, irAEs pose a significant obstacle to achieving this outcome [112, 113]. Predicting the risk of irAEs in specific patients and determining which organ the irAEs will ultimately affect is an ongoing challenge [114].

Current research focuses on explaining these phenomena from the perspectives of ICI types and host-related factors of patients [64, 115–118]. However, clinical evidence suggests that these factors account for only a portion of the observed phenomena. These differences in outcomes suggest that irAEs comprise a complex system regulated by multiple factors and that a single factor is insufficient to explain all phenomena.

Given that irAEs involve individual patient factors, tumor characteristics, and ICI types, studying tumor-related factors may provide a novel perspective. This approach completes the triangular relationship among the patient, tumor, and ICI, offering a comprehensive explanation for the aforementioned challenges.

While the occurrence and development of irAEs are associated with tumor type, the specific patterns have not yet been clearly defined [49]. The immunologic response resulting from tumor heterogeneity and patient characteristics may lead to different phenotypes of irAEs. Some irAEs can be attributed to reactions with common antigens, whereas other irAEs may represent a tumor-mediated state of tissue vulnerability, such as bystander inflammatory effects triggered by combination treatments, previous disease history, primary tumors, or metastases, where clinical immune damage has not yet emerged. In addition, tumor burden, tumor mutation burden, and gut microbiota may contribute to variations in irAEs by influencing the pattern of immune responses. Patients with different tumor types often receive distinct ICI treatments, potentially introducing biases in research outcomes [14, 119–122].

To date, no single biomarker has been shown to predict the development of irAEs in patients with cancer receiving ICI treatment, and no such biomarker has been implemented in clinical practice. Multiple factors influence the occurrence of irAEs, and it may be necessary to consider multiple factors rather than a single biomarker to effectively predict the onset of irAEs. Therefore, understanding the association between irAEs and tumors provides a novel perspective for a more comprehensive examination of adverse event mechanisms. Several questions regarding the pathophysiology of irAEs remain unanswered. As the current conclusions are primarily derived from clinical and translational studies, biospecimen collection and multi-institutional collaboration are crucial for understanding irAEs in patients. Tumors have a significant impact on irAEs, and stratified research based on tumor type should be considered in future clinical studies. The establishment of animal models for irAEs is also of great significance. Whether tumor factors should be considered during model establishment is also worth considering. As our understanding of irAE mechanisms evolves, determining which animal models, if any, best mirror organ-specific irAEs in patients will be crucial for advancing irAE research.

This review provides several important novel insights and confirms previous observations regarding irAEs. Notably, it emphasizes that different tumor types may exhibit distinct irAE patterns when treated with the same ICI therapy, a phenomenon that may become more apparent in real-world patient populations. As researchers increasingly explore expanding the therapeutic applications of ICIs and various immune activation strategies aimed at converting “cold” tumors into “hot” tumors, new challenges may emerge in the management of irAEs. A more thorough understanding of the patterns of irAEs is required to identify more accurate biomarkers in future.

## Data Availability

Data sharing is not applicable to this article, as no datasets were generated or analyzed during the current study.

## Abbreviations

ICIs: Immune-checkpoint inhibitors
irAEs: Immune-related adverse events
NK cell: Natural killer cell
PD-1: Programmed cell death protein 1
PD-L1: Programmed cell death ligand 1
IFN-γ: Interferon-γ
TME: Tumor microenvironment
NSCLC: Non-small cell lung cancer
mRCC: Metastatic renal cell carcinoma
SCC: Squamous cell carcinoma
CIP: Checkpoint inhibitors pneumonitis
